# Periodical assessment of genitourinary and gastrointestinal toxicity in patients who underwent prostate low-dose-rate brachytherapy

**DOI:** 10.1186/1748-717X-8-25

**Published:** 2013-01-30

**Authors:** Nobumichi Tanaka, Isao Asakawa, Satoshi Anai, Akihide Hirayama, Masatoshi Hasegawa, Noboru Konishi, Kiyohide Fujimoto

**Affiliations:** 1 Departments of Urology, Nara Medical University, Kashihara, Japan; 2 Department of Radiation Oncology, Nara Medical University, Kashihara, Japan; 3 Department of Pathology, Nara Medical University, Kashihara, Japan; 4 Department of Urology, Nara Medical University, 840 Shijo-cho, Kashihara, Nara, 634-8522, Japan

**Keywords:** Prostate cancer, LDR-brachytherapy, GU toxicity, GI toxicity

## Abstract

**Background:**

To compare the periodical incidence rates of genitourinary (GU) and gastrointestinal (GI) toxicity in patients who underwent prostate low-dose-rate brachytherapy between the monotherapy group (seed implantation alone) and the boost group (in combination with external beam radiation therapy (EBRT)).

**Methods:**

A total of 218 patients with a median follow-up of 42.5 months were enrolled. The patients were divided into 2 groups by treatment modality, namely, the monotherapy group (155 patients) and the boost group (63 patients). The periodical incidence rates of GU and GI toxicity were separately evaluated and compared between the monotherapy group and the boost group using the National Cancer Institute - Common Terminology Criteria for Adverse Events, version 3.0. To elucidate an independent factor among clinical and postdosimetric parameters to predict grade 2 or higher GU and GI toxicity in the acute and late phases, univariate and multivariate logistic regression analyses were carried out.

**Results:**

Of all patients, 78.0% showed acute GU toxicity, and 7.8% showed acute GI toxicity, while 63.8% showed late GU toxicity, and 21.1% showed late GI toxicity. The incidence rates of late GU and GI toxicity were significantly higher in the boost group. Multivariate analysis showed that the International Prostate Symptom Score (IPSS) before seed implantation was a significant parameter to predict acute GU toxicity, while there were no significant predictive parameters for acute GI toxicity. On the other hand, combination with EBRT was a significant predictive parameter for late GU toxicity, and rectal volume (mL) receiving 100% of the prescribed dose (R100) was a significant predictive parameter for late GI toxicity.

**Conclusions:**

The boost group showed higher incidence rates of both GU and GI toxicity. Higher IPSS before seed implantation, combination with EBRT and a higher R100 were significant predictors for acute GU, late GU and late GI toxicity.

## Background

Low-dose-rate brachytherapy (LDR-brachytherapy) is a very effective modality to administer a curative dose to the prostate while avoiding unnecessary irradiation to the normal surrounding tissues such as the urethra and rectum, especially in combination with external beam radiation therapy (EBRT), because of the achievement of a high biological effective dose (BED) [1]. On the other hand, genitourinary (GU) toxicity and gastrointestinal (GI) toxicity are important distress factors associated with LDR-brachytherapy. Many investigators have reported adverse events after LDR-brachytherapy. Usually, adverse events are evaluated in the acute and late phases using the Radiation Therapy Oncology Group (RTOG) scale [2-8] or the National Cancer Institute (NCI) Common Terminology Criteria for Adverse Events (CTCAE) [9-13]. However, there have been few reports that refer to the periodical incidence of adverse events after LDR-brachytherapy [7].

In this study, we evaluated the GU and GI toxicity in patients who underwent LDR-brachytherapy, not only in the acute and late phases, but also in each period after seed implantation using the NCI-CTCAE. We also compared the incidence rates of GU and GI toxicity in the monotherapy group (seed implantation alone) with those in the boost group (combination of external beam radiation therapy). To our best knowledge, this is the first study designed to assess the periodical incidence rates of both GU and GI toxicity in patients who underwent prostate LDR-brachytherapy.

## Methods

A total of 218 patients who were clinically diagnosed with localized prostate cancer (cT1c-2cN0M0) and who underwent LDR-brachytherapy between July 2004 and November 2008 were enrolled in this prospective study. The patients’ characteristics are shown in Table 1. The median age, PSA value at diagnosis, and follow-up period were 68.7 years (range: 51–80), 8.7 ng/mL (range: 3.1-32.1), and 42.5 months (range: 1–72), respectively. A single pathologist (K.N) with expertise in prostate cancer diagnosis reviewed the Gleason score of all biopsy specimens centrally. GU and GI toxicity were evaluated using the National Cancer Institute - Common Terminology Criteria for Adverse Events, version 3.0 (CTCAE ver.3.0) at 1, 3, and 6 months after seed implantation, and every 6 months thereafter. The incidence rates of each adverse event at 1 to 5 months, 6 to 12 months, 13 to 24 months, 25 to 36 months, and 37 to 48 months were separately calculated according to CTCAE ver. 3.0 grading, and were compared between the monotherapy group and the boost group. This study was performed in compliance with the Helsinki Declaration. The institutional reviewer board approved this prospective study, and informed consent was obtained from all patients after explaining the aim and methods of this study.

**Table 1:** Patients’ characteristics

	**Monotherapy**	**Boost**	**Total**	**p value**
	**(n = 155)**	**(n = 63)**	**(n = 218)**	
Age (year)				
mean ± SD	68.1 ± 6.6	70.3 ± 6.2	68.7 ± 6.5	0.023 ^§^
PSA at diagnosis (ng/mL)				
mean ± SD	7.2 ± 2.4	12.5 ± 6.1	8.7 ± 4.5	< 0.001 ^§^
10 or less	139	22	161	
10-20	16	34	50	
greater than 20	0	7	7	< 0.001 ^※^
biopsy Gleason score				
6 or less	111	22	133	
7	44	32	76	
8-10	0	9	9	< 0.001 ^※^
clinical T stage				
T1c	99	32	131	
T2a	51	19	70	
T2b	5	6	11	
T2c	0	6	6	< 0.001 ^※^
neoadjuvant/adjuvant ADT				
none	112	37	149	
neoadjuvant (+)	42	18	60	
adjuvant (+)	0	5	5	
both	1	3	4	0.001 ^※^
IPSS at baseline				
mean ± SD	8.7 ± 6.9	7.2 ± 5.6	8.3 ± 6.6	0.107 ^§^
Follow-up period (month)				
mean ± SD	44.2 ± 14.9	38.3 ± 16.2	42.5 ± 15.5	0.011 ^§^

^※^ Chi-square test and ^§^*t*-test.

### Treatment

Of all the patients, 149 did not receive neoadjuvant or adjuvant androgen deprivation therapy (ADT), 4 received both neoadjuvant and adjuvant ADT, 60 received only neoadjuvant ADT, and 5 received only adjuvant ADT. The study treatment was seed implantation alone in 155 patients (monotherapy group), whereas 63 patients were treated with seed implantation in combination with EBRT (boost group) (Table 1).

From July 2004 to April 2007, there were 97 patients who were treated with seed implantation at a prescribed dose of 145 Gy, and 58 patients were treated at a prescribed dose of 160 Gy after May 2007. The prescribed dose was 110 Gy for the patients who received seed implantation in combination with EBRT. The target portion of EBRT was determined one month after seed implantation, and the patients received 45 Gy (in 25 fractions of 1.8 Gy per fraction) using a four-field box technique with 6–10 MV photon energy. The clinical target volume included both the whole prostate and a third of the proximal seminal vesicle.

From July 2004 to April 2007, seed implantation was performed after preplanning by modified peripheral loading techniques using a Mick’s applicator [14]. From May 2007 to October 2008, we introduced an intraoperative planning method, and thereafter we used a real-time planning technique and a peripheral loading technique.

### Postdosimetric evaluation

Therapeutic planning and post-implant dosimetric evaluation were performed using the planning system Interplant Version 3.3 (CMS, Inc., St. Louis, USA) from July 2004 to October 2008, and Variseed 8.0 (Varian Medical Systems, Palo Alto, CA, USA) thereafter.

Post-implant CT scanning and post-implant dosimetric study was performed by a single radiation oncologist (A.I) at 1 month after seed implantation. The dosimetric parameters analyzed in this study were the minimal percentage of the dose received by 90% of the prostate gland (%D90), minimal dose (Gy) received by 90% of the prostate gland (D90), percentage of the prostate volume receiving 100% and 150% of the prescribed minimal peripheral dose (V100/150), minimal percentage of the dose and minimal dose (Gy) received by 30% of the urethra (%UD30 and UD30), rectal volume (mL) receiving 100% of the prescribed dose (R100), minimal percentage of the dose and minimal dose (Gy) received by 30% of the rectum (%RD30 and RD30),and biologically effective dose (BED). BED was calculated to evaluate an independent factor to predict GU and GI toxicity, and an α/β ratio of 2 was used [1].

### Statistic analysis

To elucidate independent factors to predict grade 2 or higher GU and GI toxicity in the acute and late phases, the prostate volume at postdosimetry, %D90, D90, V100, V150, UD30, %UD30, R100, %RD30, RD30, BED, use of neoadjuvant ADT, adjuvant ADT, International Prostate Symptom Score (IPSS), treatment modality (monotherapy vs. boost), and prescribed dose (145 Gy, 160 Gy, or 110 Gy) were evaluated.

In this study, acute toxicity was defined as toxicity that occurred < 6 months after seed implantation and late toxicity as toxicity that occurred after 6 months or later. The *t*-test was used to compare continuous variables, and the chi-square test for categorical variables.

The chi-square test was also used to test the difference in the incidence of adverse events between the monotherapy group and the boost group at each visit. Both univariate and multivariate logistic regression analyses (stepwise selection method) were conducted to discriminate the predictive parameters of grade 2 or higher GU and GI toxicity in the acute and late phases. The parameters that showed univariate significance (p-value of less than 0.10) were input into multivariate models. All statistical analyses were performed using PASW Statistics 17.0 (SPSS Inc., Chicago, IL, USA). All *p* values below 0.05 were considered statistically significant.

## Results

PSA at diagnosis, biopsy Gleason score, and clinical stage in the monotherapy group were significantly higher than those in the boost group, while patients’ age in the monotherapy group was significantly younger than that in the boost group. A higher proportion of patients in the boost group received androgen deprivation therapy compared with the monotherapy group. There were no significant differences in the baseline IPSS between the monotherapy group and the boost group. The mean follow-up period in the monotherapy group was significantly longer than that in the boost group (Table 1).

Regarding the postdosimetric parameters, %D90, V100, V150, and BED in the boost group were significantly higher than those in the monotherapy group, while UD30 and D90 (Gy) of the boost group was significantly lower than that in the monotherapy group (Table 2).

**Table 2:** Postdosimetric parameters (all patients: n = 218)

	**Monotherapy**	**Boost**	**P value**
	**(n = 155)**	**(n = 63)**	**(*t*-test)**
PV (mL) at postdosimetry	27.8 ± 8.3	27.2 ± 9.4	0.660
%D90 (%)	109.4 ± 9.7	113.7 ± 9.1	0.002
D90 (Gy)	164.7 ± 16.6	125.1 ± 10.0	< 0.001
V100 (%)	93.3 ± 3.8	94.8 ± 2.8	0.005
V150 (%)	61.5 ± 9.9	64.9 ± 11.0	0.029
UD30 (Gy)	211.1 ± 28.5	156.0 ± 20.1	< 0.001
%UD30 (%)	140.9 ± 19.0	141.7 ± 18.6	0.784
R100 (mL)	0.08 ± 0.16	0.11 ± 0.19	0.349
%RD30 (%)	26.1 ± 8.1	27.2 ± 7.4	0.330
RD30 (Gy)	39.9 ± 11.0	30.3 ± 7.4	< 0.001
BED (Gy2)	174.3 ± 18.4	216.1 ± 23.2	< 0.001

Of all patients, 78.0% showed grade 1 or higher acute GU toxicity, 7.8% showed grade 1 or higher acute GI toxicity, 63.8% showed grade 1 or higher late GU toxicity, and 21.1% showed grade 1 or higher late GI toxicity (Table 3).

**Table 3:** GU and GI toxicity in all patients

	**Grade 0**	**Grade 1**	**Grade 2**	**Grade 3**
GU (acute)	48 (22.0)	157 (72.0)	10 (4.6)	3 (1.4)
GU (late)	79 (36.2)	112 (51.4)	25 (11.5)	2 (0.9)
GI (acute)	201 (92.2)	16 (7.3)	1 (0.5)	0
GI (late)	172 (78.9)	40 (18.3)	6 (2.8)	0
NCI-CTCAE (ver. 3.0)				(%)

**Figure 1:**
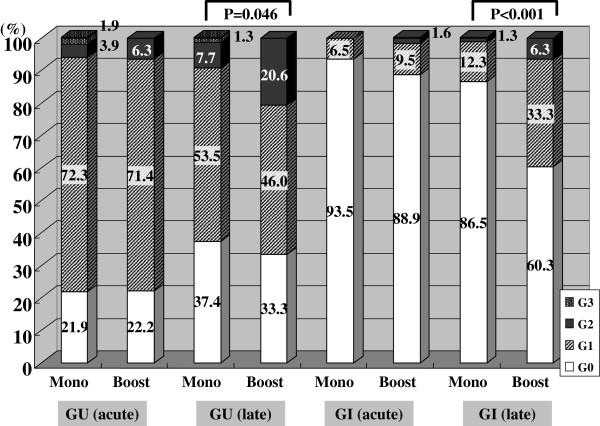
The incidence rates of both acute and late GU and GI toxicity stratified by the monotherapy group and the boost group.

Figure 1 shows a comparison of GU and GI toxicity between the monotherapy group and the boost group in both the acute and late phases. There were no significant differences in acute GU and GI toxicity between the two groups, while late GU and GI toxicity were significantly higher in the boost group.

**Figure 2:**
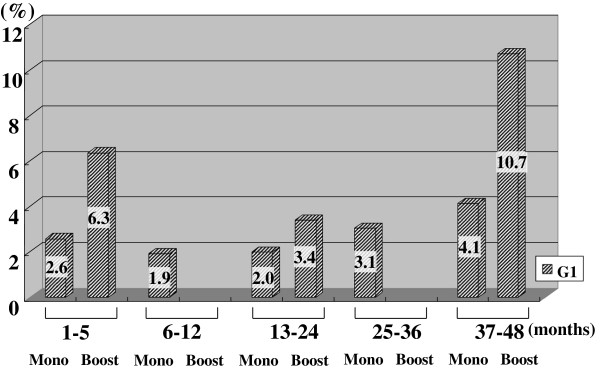
The periodical incidence rates of hematuria.

**Figure 3:**
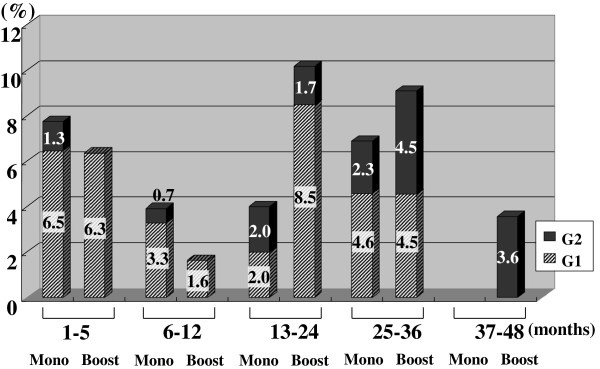
The periodical incidence rates of miction pain.

**Figure 4:**
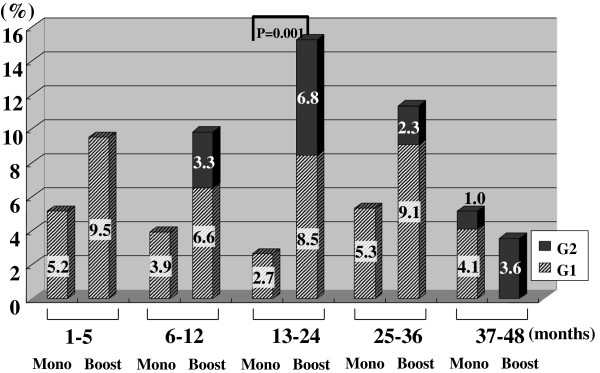
The periodical incidence rates of urinary incontinence.

**Figure 5:**
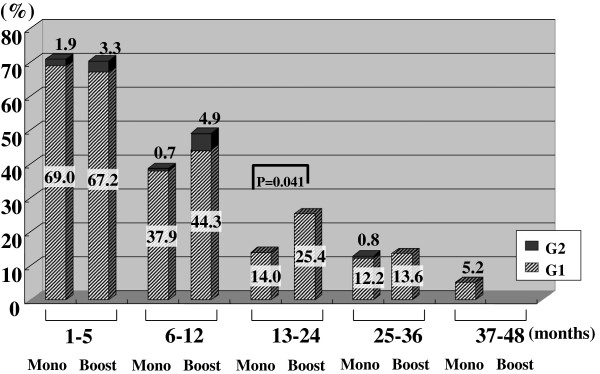
The periodical incidence rates of urinary frequency/urgency.

**Figure 6:**
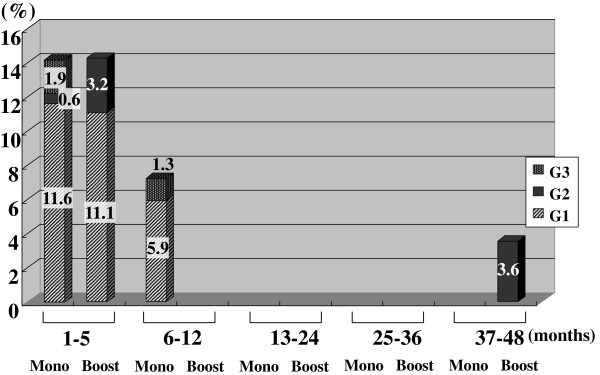
The periodical incidence rates of urinary retention.

**Figure 7:**
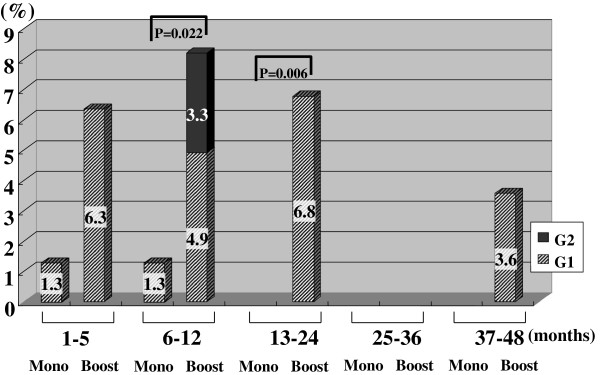
The periodical incidence rates of proctitis.

**Figure 8:**
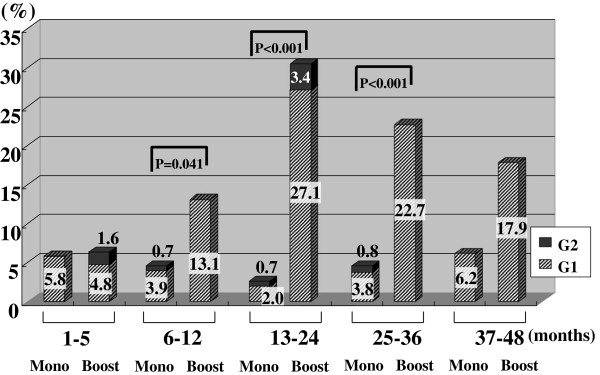
The periodical incidence rates of rectal bleeding.

Figures 2, 3, 4, 5, 6, 7 and 8 shows the results of each comparison of GU and GI toxicity regarding hematuria, miction pain, urinary incontinence, urinary frequency/urgency, urinary retention, proctitis, and rectal bleeding. Severe complications were infrequent, most complications were G2 or lower, except for urinary retention. There were several patients who developed G3 urinary retention in the monotherapy group. Overall, 8 of all the patients developed acute urinary retention during the follow-up period. Six of these 8 patients developed acute urinary retention within 1 month after seed implantation, and the other 2 developed it at 42 and 53 months after seed implantation, respectively. The incidence rates of urinary incontinence, urinary frequency/urgency rectal bleeding and proctitis in the boost group were significantly higher than those in the monotherapy group.

To elucidate the predictive parameters for GU and GI toxicity, univariate and multivariate logistic regression analyses were carried out (Table 4). IPSS before seed implantation and neoadjuvant ADT remained as predictive parameters for acute GU toxicity in the univariate analysis. In the multivariate analysis, IPSS before seed implantation was a significant predictive parameter for acute GU toxicity, and there was no significant predictive parameter for acute GI toxicity. On the other hand, BED, %D90, V100 and combination with EBRT remained predictive parameters for late GU toxicity in the univariate analysis. Finally, combination with EBRT remained in the multivariate analysis. Regarding late GI toxicity, BED, R100, %RD 30 and combination with EBRT remained in the univariate analysis. Finally, R100 remained in the multivariate analysis.

**Table 4:** The univariate and multivariate analyses in predicting acute GU, late GU and late GI toxicity of grade 2 or greater in all patients

		**Univariate**	**Multivariate**		
		**P value**	**P value**	**OR**	**95% C.I.**
*Acute GU*					
IPSS		0.025	0.025	1.084	1.010–1.163
ADT	no	reference			
	yes	0.057	n.s.		
*Late GU*					
BED (Gy2)		0.020	n.s.		
%D90 (%)		0.069	n.s.		
V100 (%)		0.088	n.s.		
EBRT	no	reference			
	yes	0.022	0.022	2.619	1.152–5.951
*Late GI*					
BED (Gy2)		0.049	n.s.		
R100 (mL)		0.02	0.034	16.626	1.235–223.837
%RD30 (%)		0.039	n.s.		
EBRT	no	reference			
	yes	0.061	n.s.		

## Discussion

Many investigators [2-14] have reported adverse events after LDR-brachytherapy. In some studies the adverse events were assessed by the RTOG or RTOG/EORTC scale [2-8], and in others, by the NCI-CTCAE scale [9-13]. Acute and late GU and GI toxicity were mostly evaluated in tetrameric style. To our best knowledge, this is the first study designed to assess the periodical incidence rates of both GU and GI toxicity in patients who had undergone prostate LDR-brachytherapy. Overall, severe adverse events were infrequent in the present study population. Only 6 patients developed grade 3 toxicity (3 patients: acute GU, 3 patients: late GU). No patients developed grade 3 GI toxicity. Stratified by treatment modality, the boost group showed significantly higher incidence rates of late GU and GI toxicity compared with the monotherapy group. On the other hand, there were no significant differences in acute GU and GI toxicity between the two groups.

In particular, acute GU toxicity was well observed in both the monotherapy group and the boost group. Around 80% of patients showed acute GU toxicity. This is comparable to the previous reports [5,7,10,11]. Our previous report has also demonstrated that an objective parameter (uroflowmetry) and a subjective parameter (IPSS) showed transient deterioration in the first 6 months after seed implantation [15]. There were no significant differences between the two groups. However, the grade of toxicity was low, and most patients had grade 1. On the other hand, acute GI toxicity was not frequently observed in either group, as was previously reported [5,9,11].

To see the details of the GU and GI toxicities in each follow-up period, there were no significant differences in the incidence rates of hematuria, urinary retention between the monotherapy and the boost groups (Figures 2, 3, 6), but the incidence rates of urinary incontinence, urinary frequency/urgency, proctitis and rectal bleeding were significantly higher in the boost group than in the monotherapy group (Figures 4, 5, 7, 8). In particular, rectal bleeding and proctitis were often observed in the boost group. There were no significant differences in the R100 value between the monotherapy and the boost groups (Table 2). It can however easily be conceived that the total dose to the rectum must be higher in the boost group. Indeed, Snyder et al. reported that the risk of developing grade 2 proctitis was significantly associated with the rectal volume (cut-off value: 1.3 mL) receiving the prescribed dose (160 Gy) [2]. Zelefsky also reported similar results [9]. Ohashi et al. also reported that the predictive parameter of grade 2 or higher GI toxicity was the maximal rectal dose in multivariate analysis [4]. Aoki et al. concluded that R150 was a significant prognostic factor for rectal bleeding in multivariate analysis [6].

In our present study, IPSS was the only prognostic factor predicting grade 2 or higher acute GU toxicity in multivariate analysis. Zelefsky et al. had similarly concluded that treatment modality (implant alone vs. combined modality) and IPSS were significant predictors of the incidence of acute grade 2 toxicities by CTCAE grading [11]. Keyes et al. also reported that the IPSS was the most significant factor by the RTOG score [7]. On the other hand, there were no significant factors predicting grade 2 or higher acute GI toxicity in our study, because there were only a few patients who developed grade 2 or higher acute GI toxicity. Meanwhile, our present study demonstrated that combination with EBRT was a predictive factor for late GU toxicity. The only predictive factor for late GI toxicity in our present study was R100. This result was comparable to previous reports [6,8,9,11].

There were several limitations in the present study such as a small number of patients (n = 218), a medium follow-up period (42.5 months), a heterogeneous patient population, etc. However, we believe that it is meaningful to assess the periodical incidence rates of both GU and GI toxicity in detail to elucidate the time course changes of urinary and rectal morbidity after seed implantation, because most previous reports had merely evaluated acute and late GU and GI toxicity in acute and late phases.

## Conclusions

The incidence rates of GU and GI toxicity were significantly different between patients in the monotherapy and the boost groups. The boost group showed a higher incidence rate, especially of GI toxicity. Patients with a higher IPSS before seed implantation showed a higher incidence rate of acute GU toxicity, while patients treated with EBRT showed a higher incidence rate of late GU toxicity. Regarding late GI toxicity, R100 was a significant parameter

## Abbreviations

LDR-brachytherapy: Low-dose-rate brachytherapy; PSA: Prostate-specific antigen; EBRT: External beam radiation therapy; IPSS: International Prostate Symptom Score; BED: Biologically effective dose; ADT: Androgen deprivation therapy; %D90: Minimal percentage of the dose received by 90% of the prostate gland; D90: Minimal dose (Gy) received by 90% of the prostate gland; V100/150: Percentage prostate volume receiving 100% and 150% of the prescribed minimal peripheral dose; %UD30: Minimal percentage of the dose received by 30% of the urethra; UD30: Minimal dose (Gy) by 30% of the urethra; %RD30: Minimal percentage of the dose received by 30% of the rectum; RD30: Minimal dose (Gy) by 30% of the rectum; R100: Rectal volume (mL) receiving 100% of the prescribed dose; PV: Prostate volume.

## Competing interests

The authors declare that they have no competing interests.

## Authors’ contributions

TN, FK, and HM conceived of this study. AI, AS, KN, and TN participated in data collection. FK and HA helped to draft the manuscript. TN carried out the statistical analysis. All authors read and approved the final manuscript.
